# Synthesis of cobalt nanoparticles on Si (100) by swift heavy ion irradiation

**DOI:** 10.1186/1556-276X-8-433

**Published:** 2013-10-18

**Authors:** Asha Attri, Ajit Kumar, Shammi Verma, Sunil Ojha, Kandasami Asokan, Lekha Nair

**Affiliations:** 1 Department of Physics, Jamia Millia Islamia, New Delhi 110025, India; 2 Inter University Accelerator Centre, Aruna Asaf Ali Marg, New Delhi 110067 India

**Keywords:** Cobalt nanostructures, Ion irradiation, Surface restructuring, Atomic force microscopy, Thin films

## Abstract

We report the growth and characterization of uniform-sized nanoparticles of cobalt on n-type silicon (100) substrates by swift heavy ion (SHI) irradiation. The Co thin films of 25-nm thicknesses were grown by e-beam evaporation and irradiated with two different types of ions, 45-MeV Li^3+^ and 100-MeV O^7+^ ions with fluences ranging from 1 × 10^11^ to 1 × 10^13^ ions/cm^2^. SHI irradiation, with the beam rastered over the area of the film, resulted in the restructuring of the film into a dense array of Co nanostructures. Surface topography studied by atomic force microscopy revealed narrowed size distributions, with particle sizes ranging from 20 to 50 nm, formed through a self-organized process. Ion fluence-dependent changes in crystallinity of the Co nanostructures were determined by glancing angle X-ray diffraction. Rutherford backscattering spectroscopy analysis showed the absence of beam-induced mixing in this system. Surface restructuring and beam-induced crystallization are the dominant effects, with the nanoparticle size and density being dependent on the ion fluence. Results are analyzed in the context of molecular dynamics calculations of electron-lattice energy transfer.

## Background

The synthesis of metallic nanoparticles from thin films offers the possibility of scientific insight into novel processes [1] as well as advantages in development due to the adaptation of existing thin film technology. Potential applications range from nanostructured magnetic media [2] to catalysts in energy conversion and templates for the growth of semiconductor nanowires and CNTs [3]. Apart from this, the plasmonic resonance in metal nanoparticles is extremely sensitive to changes in the surroundings, making them suitable for biosensing, with diagnostic and therapeutic applications being developed [4]. Today, these materials are being synthesized and modified by various physical and chemical methods. However, the synthesis of size-controlled nanoparticles remains a challenge, and many new methods are being explored. Restructuring of thin films of a few tens of nanometer thickness is one such route, which has been undertaken for a wide range of materials, with films being fabricated in various ways, *viz.*, sol–gel, spin coating [5], RF magnetron sputtering[6], pulsed laser deposition (PLD) [7], and electron beam evaporation [8]. Post-growth processing has been broadly categorized into two - thermal treatment and irradiation by particle beams. The former method involves uniform heating in vacuum [3] or laser beam irradiation resulting in either uniform heating or variable heating through the creation of thermal distribution patterns on the surface by interference of laser beams [9]. The particle beam treatment may range from the use of ion beams of a few keV from lab ion guns [10] to high-energy beams with hundreds of MeV available from ion beam particle accelerators [11,12].

Highly focused ion beam (FIB) systems offer excellent control and the possibility of 'sculpture’ on the nanoscale, but such top-down methods suffer from limitations of time, scalability, and wastage of resources [1]. Ion beam irradiation is a unique tool for engineering nanoparticles as it provides the possibility of both 'top-down’ and 'bottom-up’ mechanisms to produce nanoparticles of uniform sizes, using the intrinsic properties of the metals and metal-substrate interfaces. As the incident ion passes through a solid, energy is lost via interactions with target nuclei (nuclear energy loss (Sn)) and target electrons (electronic energy loss (Se)) [13]. In the low-energy ion irradiation regime (up to a few tens of keV), collisions with target nuclei are significant, with atomic displacements and recoiled target atoms yielding collision cascades. Upon irradiation by what are called swift heavy ions (SHI; or ions of masses greater than H and He, with energies in the MeV region), electronic energy loss is dominant, with ionization along the ion track and significant energy transfer to the electrons of the target. This energy is then transferred to the lattice, resulting in a 'thermal spike,’ as the kinetic energy of the atoms along the ion track goes up dramatically for a few fractions of a picosecond following the ion transit. This could lead to significant atom displacement and mass movement, even to the surface [14]. The resulting changes in the target depend on the systems being considered (ion mass as compared to the target, ionic charge, incident energy, and the electronic structure of the target - whether metal, semiconductor, or insulator), varying from amorphization to crystallization and shape change of implanted targets in the direction of the ion beam [15,16]. In the case of metal films, due to the delocalized electronic configuration, models of energy transfer have had to consider electronic thermal spike effects as well [17-19].

Co nanoparticles are important due to crystallite size-dependent magnetic properties and site-selective catalytic activity [19,20]. Some irradiation studies on the same Co/Si (100) system have been reported in the literature, with results that vary considerably depending on the identity and energy of the incident ions. Lian et al. [1] used a 25-keV FIB beam for initial processing to form strips of various widths and then rastered the defocused beam across the film, resulting in sputtering and dewetting of the film, driven by the Rayleigh instability [1]. Agarwal et al. [21] have found silicide formation at the interface of the Co/Si system upon irradiation with much heavier Au ions of 120 MeV. Mixing at the interface has also been reported with this system [22,23], with Rutherford backscattering spectroscopy (RBS) being used to quantify the changes observed.

Our work is an effort to characterize the change in morphology observed upon irradiation with ions of different masses and incident energies, and the effect of ion fluence on the development of nanoparticles so as to gain insight into the mechanism by which such large-scale atom movement occurs.

## Methods

The samples were prepared at the Target Lab of the Inter University Accelerator Centre, New Delhi (IUAC). A Co thin film of 25-nm thickness was deposited on n-type silicon (100) substrates with a size of approximately 1 × 1 cm^2^ (scored and cut from commercial wafers and cleaned by sonicating in warm acetone and methanol) by e-beam evaporation at room temperature. Vacuum during deposition was maintained at approximately 7 × 10^-7^ mbar, and a quartz crystal monitor was used to monitor the thickness of the deposited film. These films were later irradiated with ions from the 15 UD Pelletron accelerator at the IUAC. In order to characterize the ion type, energy, and fluence required for nanostructure formation, these films were bombarded with Li^3+^ and O^7+^ beams, having an energy of 45 and 100 MeV, respectively. For both sets of ions, the samples were irradiated with five different fluences, i.e., 1 × 10^11^, 5 × 10^11^, 1 × 10^12^, 5 × 10^12^, and 1 × 10^13^ ions/cm^2^. We present data from three sets of fluences, i.e., 1 × 10^11^, 1 × 10^12^, and 1 × 10^13^ ions/cm^2^, for clarity.

When the energetic ions collide with the target atoms of the deposited Co film, there are two types of energy loss mechanisms - nuclear energy loss (dominant in the keV energy range) and electronic energy loss (which is dominant in the MeV energy range). In our experiment, 45-MeV Li^3+^ and 100-MeV O^7+^ ions have nuclear energy loss of 0.1762 and 1.224 keV/μm, respectively, and electronic energy loss of 3.098 × 10^2^ and 2.076 × 10^3^ keV/μm, respectively. Li^3+^ and O^7+^ ions have projected ranges of 88.95 and 34.10 μm, respectively in Co/Si. Thus, the loss mechanism in the given energy range is dominated by electronic energy loss in both cases, but the loss in the case of oxygen ions is about seven times higher, allowing for a comparative study. As mentioned above, reports in the literature also indicate that the mass of the ion has a significant effect on the consequences of ion bombardment [21-23]. The above parameters were obtained on the basis of SRIM-2008 calculations [24].

Surface structure and roughness were analyzed by atomic force microscopy (Nanoscope IIIa (Veeco, Santa Barbara, CA, USA) at the Material Science Group, IUAC) in the tapping mode with an antimony-doped silicon tip. The crystal structure is studied by X-ray diffraction (XRD) carried out using a PW3710 diffractometer (Phillips, Eindhoven, Netherlands; at the Crystal Growth Laboratory, Department of Physics, Jamia Millia Islamia, New Delhi) with nickel-filtered Cu Kα (*λ* = 1.5418 Å); the sample was scanned over 10° to 80° at the rate of 2°/min. The composition of the as-deposited and irradiated samples is measured by Rutherford back scattering spectroscopy (RBS) using a He^+^ beam of 2-MeV energy at the Inter University Accelerator Centre [25].

## Results and discussion

### AFM results

**Figure 1:**
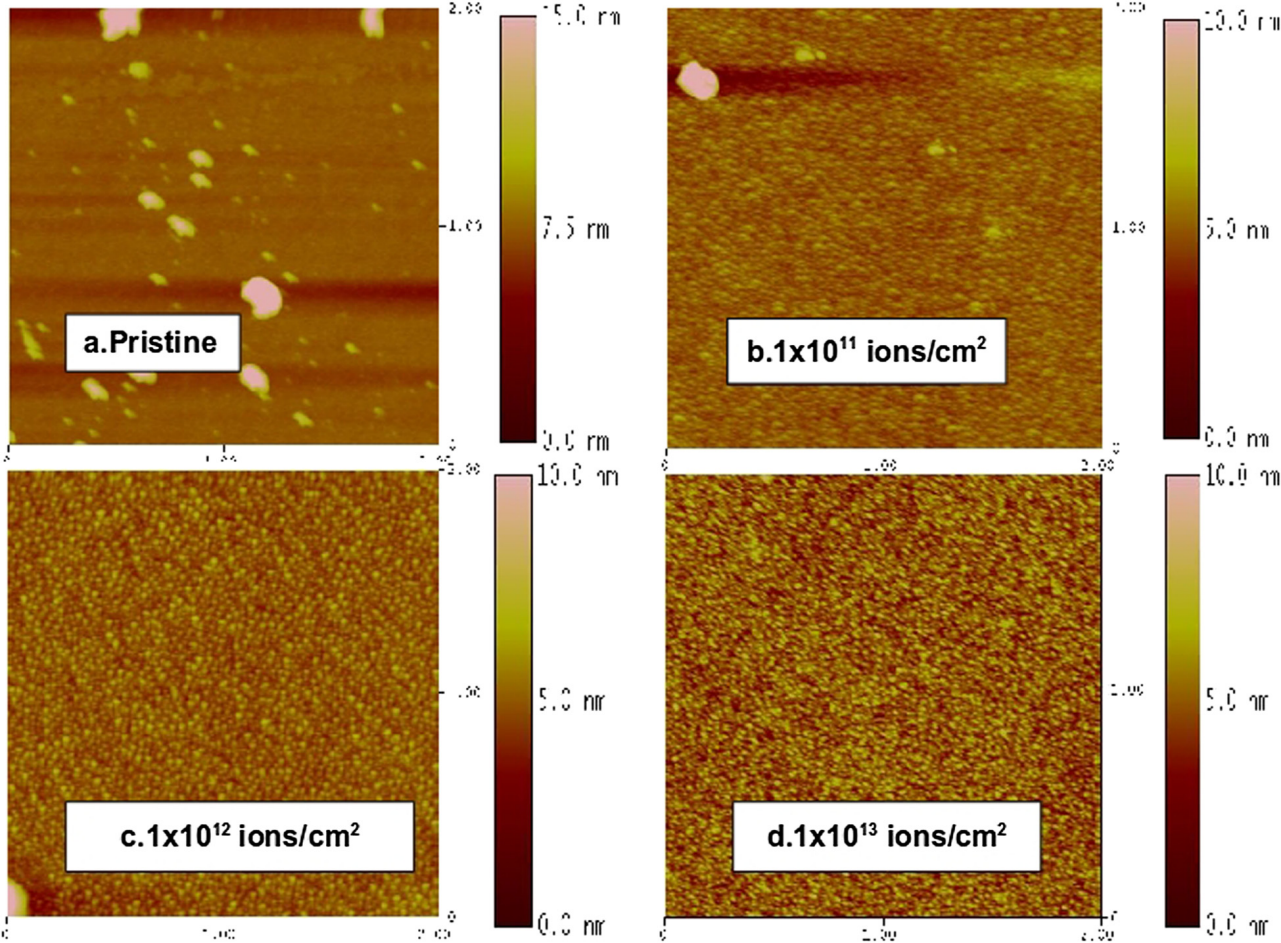
**AFM images showing 2-μm scans of the topography of pristine and O**^**7+-**^**irradiated samples, with increasing fluence. (a)** Pristine, **(b)** 1 × 10^11^ ions/cm^2^, **(c)** 1 × 10^12^ ions/cm^2^, and **(d)** 1 × 10^13^ ions/cm^2^.

Figure 1 shows the changes in surface structure from atomic force microscopy (AFM) scans of the Co films, with increasing O^7+^ fluence. Before irradiation, the surface of the pristine film is smooth and featureless on the 2-μm scale, as in Figure 1a. The scans shown are representative, as other areas on the pristine samples had similar smooth morphology. These are raw images, without digital smoothing. The large, clearly defined patches, we believe, are dust particles that may have settled on the samples during the transfer process, since the films were transferred outside vacuum into the AFM facility without further cleaning.

As irradiation proceeds, uniformly distributed nanoparticles develop, with increasing number density (particles per unit area) as fluence is increased. The grain size distribution is found to narrow with increase in ion fluence, and the shape of the grains also appears to be more well defined and uniform at the highest fluence used (see Figures 1b,c,d). Surface roughness (defined as rms deviation from the mean surface height) initially increases from a value of 0.214 nm for the pristine film to 0.650 nm for our initial fluence of 1 × 10^11^ ions/cm^2^, due to the perturbations that are created as the ions impinge on the surface. At this fluence, only 1 in 1,000 atoms on the surface has undergone a collision with an incident ion. As irradiation proceeds, a higher fraction of surface atoms is bombarded and the density of perturbed regions of the film increases and the roughness decreases until the lowest value of 0.429 nm is reached, for a fluence of 1 × 10^13^ ions/cm^2^. This roughness value is still twice that of the pristine surface, indicating the transformation that has taken place. The reduction in roughness has also been reported by Lian et al. [1] as a 'nanosmoothing’ process.

**Figure 2:**
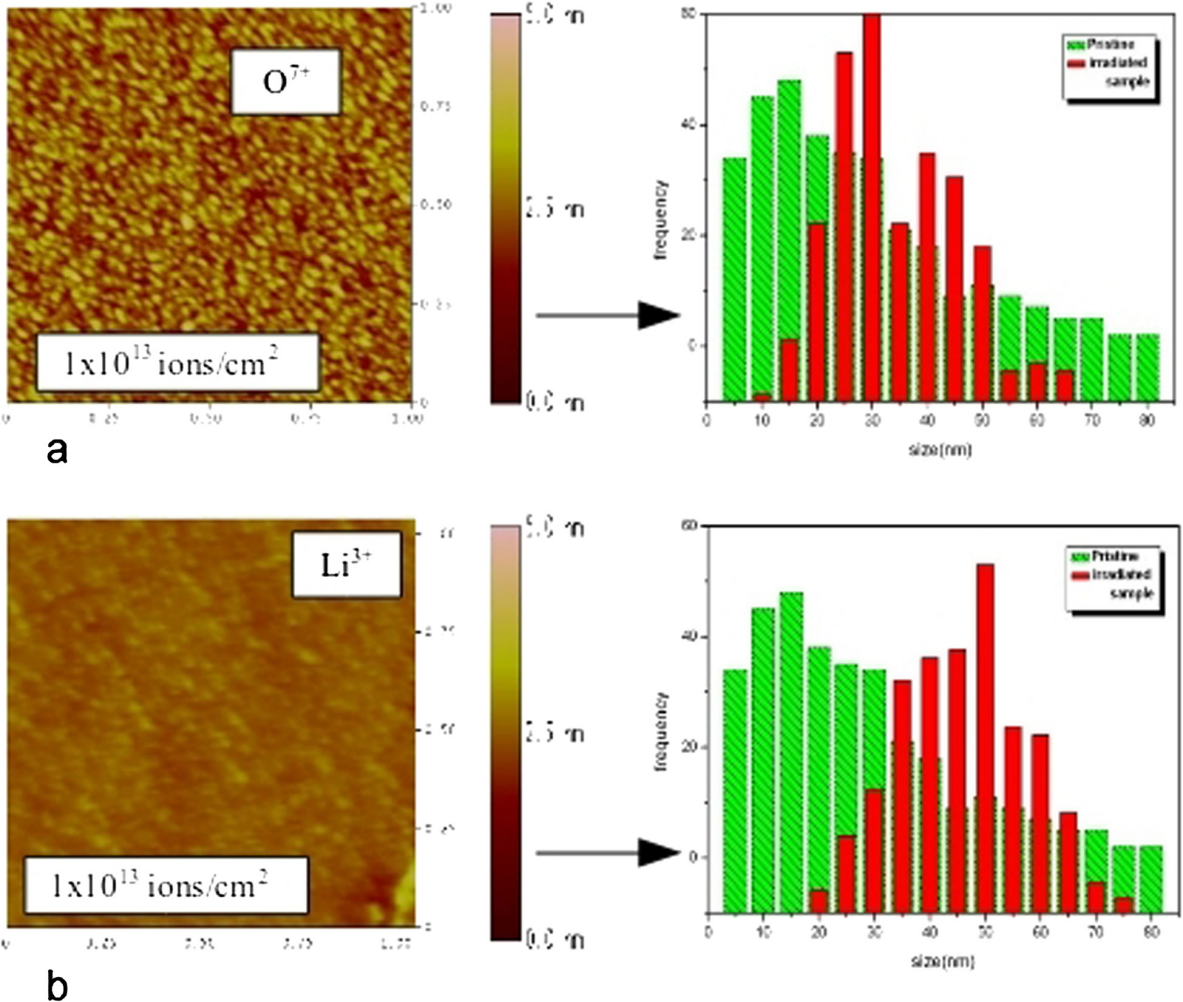
**AFM images of irradiated samples with their corresponding size distributions obtained from surface profiles.** Samples irradiated by **(a)** O^7+^ and **(b)** Li^3+^ ions.

The AFM scans are analyzed in detail in Figure 2, which shows higher resolution images (1-μm scans) obtained after irradiation with the highest fluence of 1 × 10^13^ ions/cm^2^, for both sets of incident ions, with their corresponding size distributions. The size distribution histograms were obtained from surface profiles of the images. The base width of the features on the pristine surface shows a widely spread out distribution, from 5 to 80 nm, indicating a random surface profile size (green graph). This changes for the surface in Figure 2a, which is O^7+^ irradiated (red graph): the particle size distribution narrows and has a peak at the 25- to 30-nm base width, with most of the particles having a base width in the 20- to 50-nm range. This indicates the development of a characteristic size for nanoparticles in this system.

In Figure 2b, the topography indicates that upon irradiation with the Li^3+^ beam, the nanostructures obtained are not as well defined as with the oxygen beam. The size distribution also peaks at a higher value of about 50 nm, as compared to the case of the O^7+^ beam.

**Figure 3:**
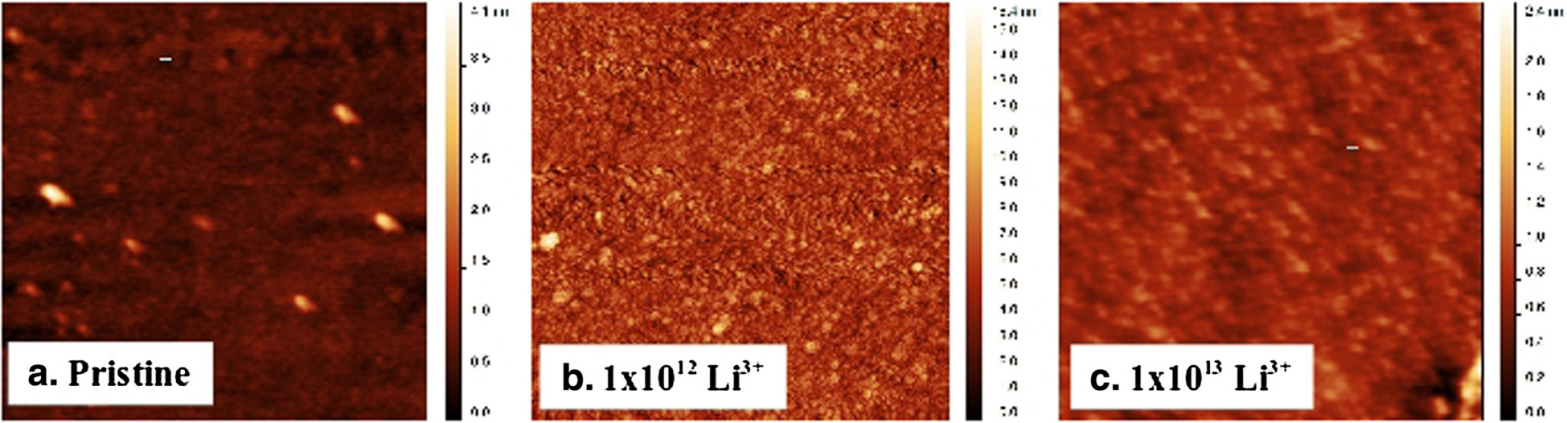
**AFM images showing 1-μm scans of the topography of pristine and Li**^**3+**^-**irradiated samples, with increasing fluence. (a)** Pristine, **(b)** 1 × 10^12^ ions/cm^2^, and **(c)** 1 × 10^13^ ions/cm^2^.

**Figure 4:**
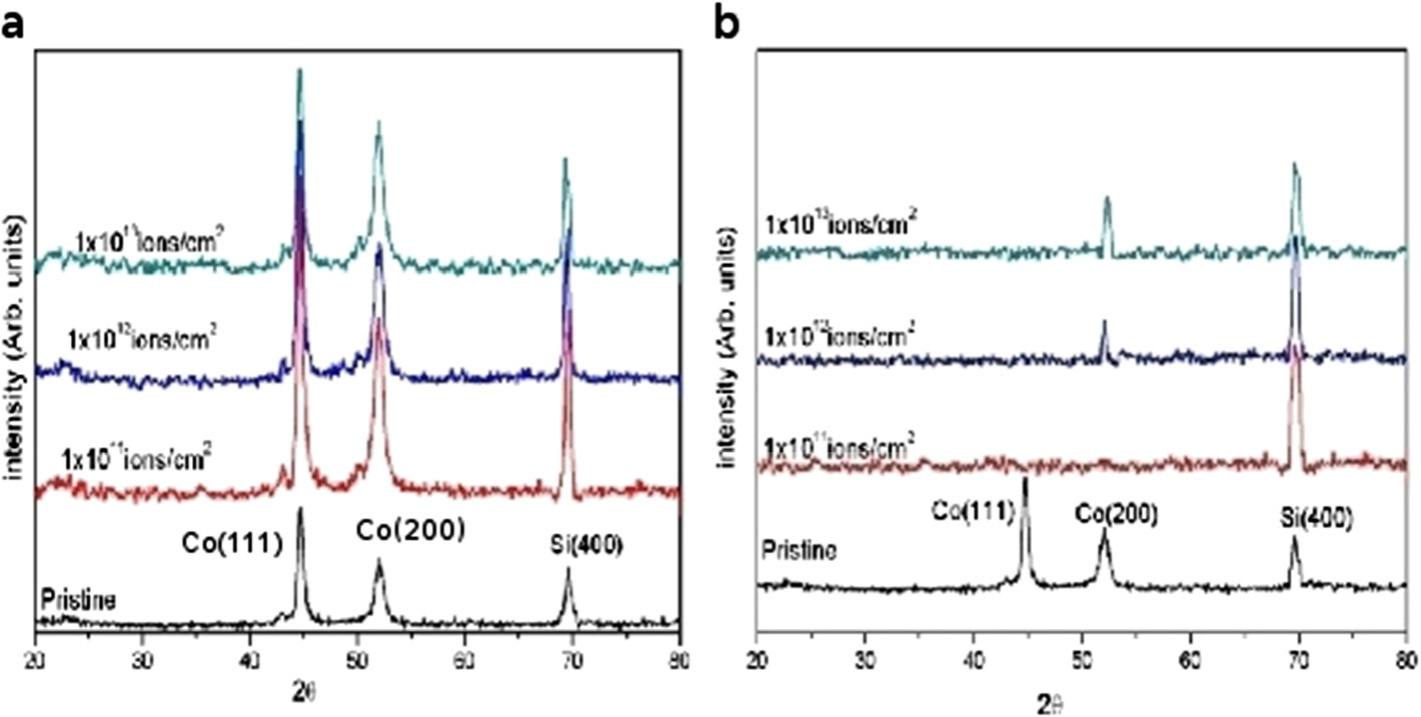
Glancing incidence XRD scans of (a) oxygen- and (b) lithium ion-irradiated samples, respectively.

Figure 3 shows more detailed images, on the 1-μm scale, of the sequence of Li-irradiated films. The morphological changes are markedly different here, as the surface structure shows even smaller features at the fluence of 1 × 10^12^ ions/cm^2^, which we have found from the profile analysis to be predominantly of the 10- to 25-nm base width, which transform into the broader structures seen at the 1 × 10^13^ ions/cm^2^ fluence value. This correlates with the XRD data for the Li-irradiated samples in Figure 4b below, which show that following initial amorphization, a crystalline Co peak reappears at the fluence of 1 × 10^12^ ions/cm^2^. As mentioned in the experimental section, the electronic energy loss value obtained upon irradiation with the O^7+^ beam is 2.076 × 10^3^ keV/μm, almost seven times that of the Li^3+^ beam (3.098 × 10^2^ keV/μm), and this would lead to a significant reduction, in the case of Li^3+^ irradiation, in the electronic energy available for transfer to the lattice through the electron–phonon coupling mechanism, which is responsible for the thermal spike in metals. This can have a significant effect on the kind of morphological changes that are created in the Co film, because thermal spike model calculations have shown that the track radii (the perturbed region where the spike has occurred) in metals increase with the electronic energy loss values [26]. The temperature of the thermal spike created by the incident ions cannot be directly measured, and estimates can only be obtained with molecular dynamics simulations which need to be done for each ion type independently [27,28]. Our results suggest that the temperature spike in the case of the lithium ion irradiation is not significant enough to cause substantial recrystallization of the film which has undergone amorphization, as indicated in the XRD data in Figure 4b.

### XRD studies

The structural modification of the Co/Si (100) system under 100-MeV oxygen and 45-MeV lithium ions has been examined by XRD. Spectra for pristine and irradiated samples are shown in Figure 4.

The pristine sample shows two peaks at 44.77° and 52.01° which are identified as cobalt (111) and (200), from the JCPDS database (JCPDS 15–0806). After irradiation with 100-MeV oxygen ions, the peak intensity increases sharply, with the increase being maximum for the lowest fluence studied. The increment in the (111) peak is seen to be more than that of the (200) peak. As SHI irradiation helps to release the strain by local heating of the film, this could be responsible for the initial improvement in crystallinity. When the film is irradiated with Li^3+^ ion at lower fluence (1 × 10^11^ ion/cm^2^), the Co peaks disappear, but the (200) peak reappears upon further irradiation at the fluences of 1 × 10^12^ and 1 × 10^13^ ions/cm^2^ (at the angle of 52.01°). The clear variation in the beam-induced effects with different ions suggest that the mechanism for the changes in crystallinity of the films depends on the specific energy and identity of the bombarding ions and can only be determined following detailed simulations of this system, as initiated in the molecular dynamics calculations reported by Mookerjee et al. [28]. Their calculations indicate the rapid quenching of the instantaneous temperature spike (up to thousands of degrees Kelvin) in the sub-picosecond time scale, so the possibility of nanoscale melting and recrystallization must be considered [29].

### RBS studies

**Figure 5:**
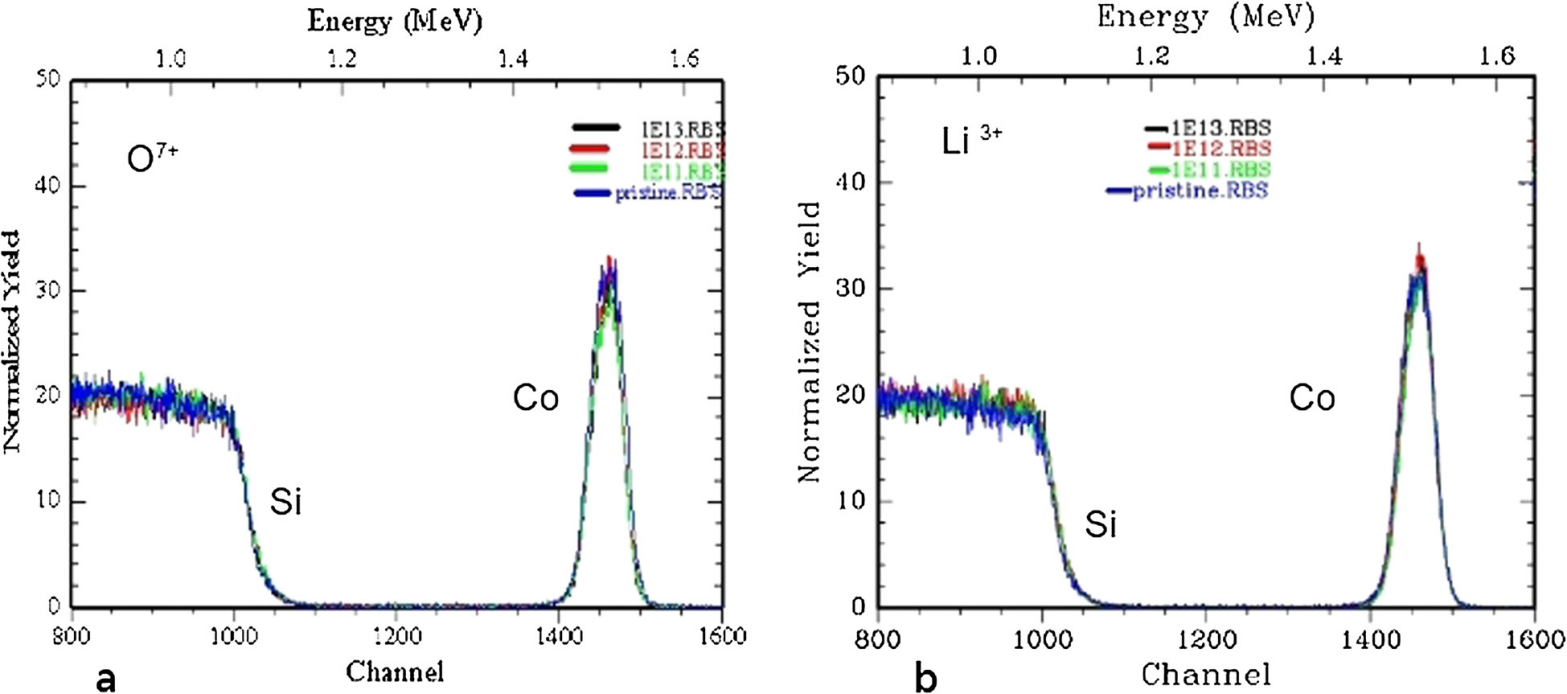
RBS data from pristine and (a) oxygen-irradiated samples and (b) lithium-irradiated samples.

The RBS data from the oxygen- and lithium-irradiated samples are shown in Figure 5. RBS studies show the elemental composition of the samples, with depth resolution. The peak at the 1.527-MeV energy confirms the presence of Co on the Si substrate. The measured thickness of the film was estimated as 25 nm by RBS as well. The depth profile of Co films was calculated by fitting the experimental RBS data using the Rutherford Universal Manipulation Programme (RUMP) simulation code. The Co peak shape and height are unchanged within the RBS resolution of 5 nm, with no evidence of sputtering, mixing, or diffusion of Co into the Si matrix. This indicates that the beam-induced changes are purely morphological, with the film rearranging itself into nanoparticles in a self-organized process. This is consistent with our XRD studies, which show no evidence of cobalt silicide peaks in this system. Other studies have reported silicide formation [21], but they have used much heavier Au ions, which could have overcome the threshold for silicide formation.

**Figure 6:**
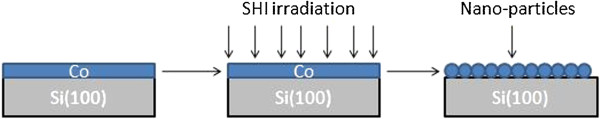
Schematic diagram of nanoparticle synthesis by SHI irradiation.

The process by which synthesis of cobalt nanoparticles is taking place can be outlined as indicated in the schematic in Figure 6 below.

Results obtained from our studies show that the synthesis of cobalt nanoparticles is strongly dependent on the mass and energy of the incident ions. AFM scans reveal that nanoislands of cobalt are formed on the n-silicon (100) surface when irradiated both with oxygen and lithium ions, but the details of the morphology obtained differ in each case. XRD analysis shows some changes in crystallinity of the Co but no evidence of silicide formation at the interface.

In the present case, the mechanism of formation of Co nanoparticles on the substrate follows from the thermal spike model [14] which suggests that a transient nanoscale local hot molten zone of several thousands of degrees Kelvin is formed along the ion track through the film. Within the picosecond time frame, the track cools down and agglomerates of metal nanoparticles are formed. Sprouster et al. reported 120-MeV Au ion-induced transformation of shape in embedded Co nanoparticles above a threshold size parallel to the incident ion directions [20]. In their case, the shape transformation is constrained by the matrix within which the Co particles are placed. In the case of metallic thin films deposited on substrates, the surface energy mismatch drives the transformation due to the local heating and leads to atomic displacement and the eventual coalescence of the film into nanoscale structures.

## Conclusion

Cobalt nanoparticles were synthesized on a silicon (100) wafer by SHI irradiation, with 45-MeV Li^3+^ and 100-MeV O^7+^ ions, at room temperature. AFM studies showed the formation of globule-shaped cobalt nanoparticles on the surface of the film. Characterization by RBS and XRD studies suggests that the crystallinity of the Co is increased and that no silicide formation was detected in our experiment. The present study suggests that SHI-induced irradiation is a promising way to grow nanoparticles from thin films through a self-organized process and that this approach enables size-controlled production of uniformly distributed nanoparticles.

## Abbreviations

AFM: Atomic force microscopy; FIB: Focused ion beam; RBS: Rutherford backscattering spectroscopy; SHI: Swift heavy ion; XRD: X-ray diffraction.

## Competing interests

The authors declared that they have no competing interests.

## Authors’ contributions

AA and AK prepared the samples and performed the irradiation and characterization. SV and SO were involved in the performance and analysis of the RBS data. AA, KA, and LN were involved in the design of the experiment, data analysis, and preparation and revision of the manuscript. All authors read and approved the final manuscript.
